# Lichen planus des Ösophagus: Eine prospektive, interdisziplinäre, monozentrische Kohortenstudie

**DOI:** 10.1111/ddg.15808_g

**Published:** 2025-11-14

**Authors:** Rebecca Diehl, Annette Schmitt‐Graeff, Dimitra Kiritsi, Wolfgang Kreisel, Arthur Schmidt, Annegrit Decker, Franziska Schauer

**Affiliations:** ^1^ Klinik für Dermatologie und Venerologie Universitätsklinikum Freiburg Medizinische Fakultät Albert‐Ludwigs‐Universität Freiburg Freiburg Deutschland; ^2^ Albert‐Ludwigs‐Universität Freiburg Freiburg Deutschland; ^3^ First Department of Dermatology and Venereology Medizinische Fakultät Aristotle University Thessaloniki Thessaloniki Griechenland; ^4^ Klinik für Innere Medizin II Gastroenterologie Hepatologie Endokrinologie und Infektiologie Universitätsklinikum Freiburg Medizinische Fakultät Albert‐Ludwigs‐Universität Freiburg Freiburg Deutschland

**Keywords:** Bolus‐Obstruktion, Denudation, Dysphagie, Plattenepithelkarzinom, Trachealisierung, bolus obstruction, denudation, dysphagia, squamous cell carcinoma, trachealization

## Abstract

**Hintergrund und Zielsetzung:**

Der Lichen planus (LP) ist eine entzündliche Erkrankung, die Haut, Schleimhäute, sowie Haarfollikel und Nägel betrifft. Die ösophageale Beteiligung ist eine unterschätzte Manifestation.

**Patienten und Methodik:**

In dieser prospektiven Kohortenstudie (2020–2023) wurden 562 Patienten auf einen symptomatischen und klinisch relevanten ösophagealen LP (ÖLP) untersucht, wobei die Dysphagie als primäres Screening‐Kriterium verwendet wurde. Die Studie umfasste Patienten mit neu diagnostiziertem oder bestehendem LP aus der Dermatologie, die über ösophageale Symptome berichteten, sowie Patienten aus der Gastroenterologie, die sich einer Endoskopie bei ungeklärten ösophagealen Beschwerden unterzogen, die prinzipiell zu einem ÖLP passend waren. Die Diagnose des ÖLP basierte auf endoskopischen, histopathologischen und Immunfluoreszenz‐Befunden.

**Ergebnisse:**

Von insgesamt 77 eingeschlossenen Patienten mit Dysphagie und potenziellem ÖLP konnte bei 21 Patienten ein ÖLP gesichert werden. Diese Patienten wiesen signifikant höhere Raten an ösophagealer Dysphagie, Nahrungsmittelbolus‐Obstruktion und retrosternalen Schmerzen im Vergleich zu Patienten ohne ÖLP auf. Bei zwei ÖLP‐Patienten wurde ein ösophageales Plattenepithelkarzinom diagnostiziert. Multilokuläre LP‐Manifestationen können ein Indikator für eine ösophageale Beteiligung sein.

**Schlussfolgerungen:**

Diese Studie unterstreicht die Notwendigkeit zur endoskopischen und dermatologischen Untersuchung bei Vorliegen einer Dysphagie bei Patienten mit LP zur Sicherung einer ösophagealen Beteiligung und Ausschluss von Metaplasien beziehungsweise Plattenepithelkarzinomen.

## EINLEITUNG

Der Lichen planus (LP) ist eine weit verbreitete entzündliche Erkrankung, die etwa 1,3% der Weltbevölkerung betrifft, mit einer höheren Inzidenz bei Frauen.^1^, ^2^, ^3^, ^4^ Der LP ist durch eine Interface‐Dermatitis gekennzeichnet, die durch eine Typ‐1‐Immunantwort ausgelöst wird und zur Nekroptose von Keratinozyten führt.^5^ Ein wichtiges Zytokin dieses Prozesses ist Interferon (IFN)‐γ, das von zytotoxischen T‐Zellen produziert wird und über den JAK/STAT‐Signalweg auf Keratinozyten wirkt.^6^

Der LP tritt in drei Hauptformen auf: Befall der Haut, der Schleimhäute und der Hautanhangsgebilde. Die Manifestationen können einzeln oder in Kombination auftreten.^3^ Der mukosale Lichen planus kann den Mund, Rachen, Speiseröhre, Genitalbereich und in seltenen Fällen die Augen betreffen. Eine orale Beteiligung ist besonders häufig und tritt bei etwa zwei Dritteln der Fälle mit Hautbeteiligung auf.^7^, ^8^ Es gibt Hinweise auf einen Zusammenhang zwischen oralem Lichen planus (OLP) und Infektionen mit dem Hepatitis‐B‐Virus (HBV) oder dem Hepatitis‐C‐Virus (HCV), die potenziell als Auslöser wirken können.^9^ Patienten mit OLP haben aufgrund der chronischen Entzündung ein erhöhtes Risiko, ein orales Plattenepithelkarzinom (PEK) zu entwickeln.^10^

Der ösophageale Lichen planus (ÖLP) ist eine von mehreren entzündlichen Erkrankungen der Speiseröhre, die bei Patienten mit ösophagealer Dysphagie in Betracht gezogen werden sollten.^11^, ^12^ Die Differenzialdiagnose für dieses Symptom ist breit gefächert und umfasst häufige Erkrankungen wie Refluxösophagitis (GERD), infektiöse Ursachen, Malignome und immunologische Störungen wie eosinophile Ösophagitis (eosinophilic esophagitis; EoE) oder blasenbildende autoimmune Erkrankungen.^13^, ^14^, ^15^, ^16^, ^17^ Obwohl in der Literatur Fälle eines ÖLP ohne extraösophageale Manifestationen eines LP beschrieben wurden, zeigen die meisten Patienten mit klinisch relevanten und behandlungsbedürftigen ÖLP mindestens eine weitere mukokutane Manifestation, wie beispielsweise eine Nagelbeteiligung.^18^

Der ösophageale Lichen planus galt lange als seltene Erkrankung, könnte aber aufgrund fehlender spezifischer diagnostischer Kriterien innerhalb des breiten Spektrums der Differenzialdiagnosen unterdiagnostiziert worden sein. In den letzten Jahren wurden diagnostische Kriterien vorgeschlagen und auf frühere klinische Kohorten angewendet.^19^, ^20^ Während einige neuere Studien darauf hindeuten, dass eine Ösophagusbeteiligung bei bis zu der Hälfte der Patienten mit kutanem LP oder OLP auftreten kann, könnte die tatsächliche Häufigkeit von ÖLP aufgrund kleiner Stichprobengrößen und möglicher Selektionsverzerrung auch niedriger sein als berichtet.^21^, ^22^, ^23^ In dieser Studie haben wir die ösophageale Beteiligung des LP prospektiv und multidisziplinär untersucht, um klinische und diagnostische Merkmale weiter zu etablieren.

### Patienten und Methodik

Von Januar 2020 bis Dezember 2023 führten wir eine prospektive Kohortenstudie in den Abteilungen für Dermatologie und Gastroenterologie am Universitätsklinikum Freiburg durch. Die Patienten wurden über zwei Wege rekrutiert: über die Dermatologie, wenn ein bestätigter Lichen planus der Haut oder Hautanhangsgebilde und Dysphagie (oropharyngeal und ösophageal) vorlag, oder über die Gastroenterologie, wenn sie zur endoskopischen Abklärung einer zuvor nicht definierten Ösophaguserkrankung überwiesen wurden, die mit einem ÖLP vereinbar sein könnte. Nach Einholung der Einwilligung wurden personenbezogene Daten erhoben. Außerdem wurden mithilfe von Fragebögen detaillierte Informationen zu den Ösophagussymptomen erfasst, darunter Dysphagie, Sodbrennen, Bolusereignisse, Regurgitation und ungewollter Gewichtsverlust.

Die dermatologische Untersuchung erfasste die Lokalisation des LP: oral, genital, anal, Auge, Haut und Nägel. Eine Endoskopie wurde durchgeführt und anhand definierter makroskopischer Kriterien analysiert (Tabelle 1).^20^ Für die histopathologische Analyse wurden Gewebeproben sowohl aus dem unteren als auch aus dem oberen Drittel der Speiseröhre entnommen. Um Interferenzen mit möglichen histopathologischen Merkmalen einer koexistierenden GERD zu minimieren, wurden keine Biopsien innerhalb von etwa 5 cm des gastroösophagealen Übergangs oder aus Bereichen mit refluxbedingten Läsionen entnommen. Zusätzlich wurde eine dritte Biopsie aus der mittleren Speiseröhre für die direkte Immunfluoreszenzmikroskopie (DIF) entnommen. Gewebeproben für die Standard‐Histopathologie wurden in Formalin fixiert, in Paraffin eingebettet und mit Hämatoxylin‐Eosin (HE), Giemsa und Periodsäure‐Schiff (PAS) gefärbt. Die DIF wurde an Dünnschnitten von frisch gefrorenem Gewebe unter Verwendung von Antikörpern gegen IgG, IgA, IgM, C3c und Fibrinogen nach einem etablierten Protokoll durchgeführt.^20^ Für die Diagnose ÖLP verwendeten wir die zuvor vorgeschlagenen diagnostischen Kriterien (Tabelle 1). Zusätzlich wurde ein serologisches Screening auf HBV‐ und HCV‐Infektion durchgeführt.

**TABELLE 1:** Diagnostische Kriterien und Schwereeinteilung des ÖLP, wie zuvor beschrieben.^20^

Makroskopische Zeichen in der Endoskopie (D als spezifisches und H, T, S als unspezifische Zeichen)		
*Denudation der Mucosa (D)*: D1: iatrogen D2: spontan (< 1 cm^2^) D3: spontan (> 1 cm^2^)	*Hyperkeratose (H*) *Trachealisierung (T)*	*Stenose (S)*: S1: endoskopisch passierbar S2: endoskopisch nicht passierbar oder Durchmesser < 1 cm
**Mikroskopische histologische Kriterien (HP): Ein Punkt pro Kriterium: HP0 (negativ) bis HP3 (stark positiv**)		
Epithelablösung Lymphozyteninfiltrat Intraepitheliale Apoptose (Civatte‐Körperchen) Dyskeratose		
**Direkte Immunfluoreszenz**		
*Fibrinogen (F)* F0: kein F1: schwach positiv F2: positiv		
**Schwereeinteilung für ÖLP**		
*Schwerer ÖLP*: ≥ D2 und HP ≥ 1 und/oder F ≥ 1 *Milder ÖLP: 1) D1 und HP ≥ 1 und/oder F ≥ 1 2) S, H, T oder kein endoskopisches Zeichen und HP1 ≥ + F ≥ 1* *Kein ÖLP: kein oben genanntes Kriterium erfüllt*		

*Abk*.: D, Denudation; DIF, direkte Immunfluoreszenz; F, Fibrinogenablagerung in der DIF; H, Hyperkeratose; HP, Histopathologie; S, Stenose; T, Trachealisierung

REDCap, eine sichere webbasierte Plattform der Universität Freiburg, ermöglichte eine standardisierte und systematische Datenerfassung für die Patientenkohortenforschung.^24^, ^25^ Die Daten wurden anonymisiert mit der R‐Studio‐Software Nr. 2024.04.1+748 analysiert. Der t‐Test wurde verwendet, um numerische und binäre Variablen zu vergleichen. Der exakte Fisher‐Test wurde angewendet, um binäre und gruppierte Variablen zu vergleichen. P‐Werte < 0,5 wurden als statistisch signifikant angesehen. Die Studie wurde von der Ethikkommission in Freiburg mit der Nummer 20‐1227‐1 genehmigt. Die Studie wurde im Deutschen Register Klinischer Studien (DRKS, DRKS00023700) registriert. Das Manuskript wurde gemäß der STROBE‐Checkliste strukturiert.^26^

## ERGEBNISSE

### Demografische Daten und Charakterisierung der Gruppen

**ABBILDUNG 1:**
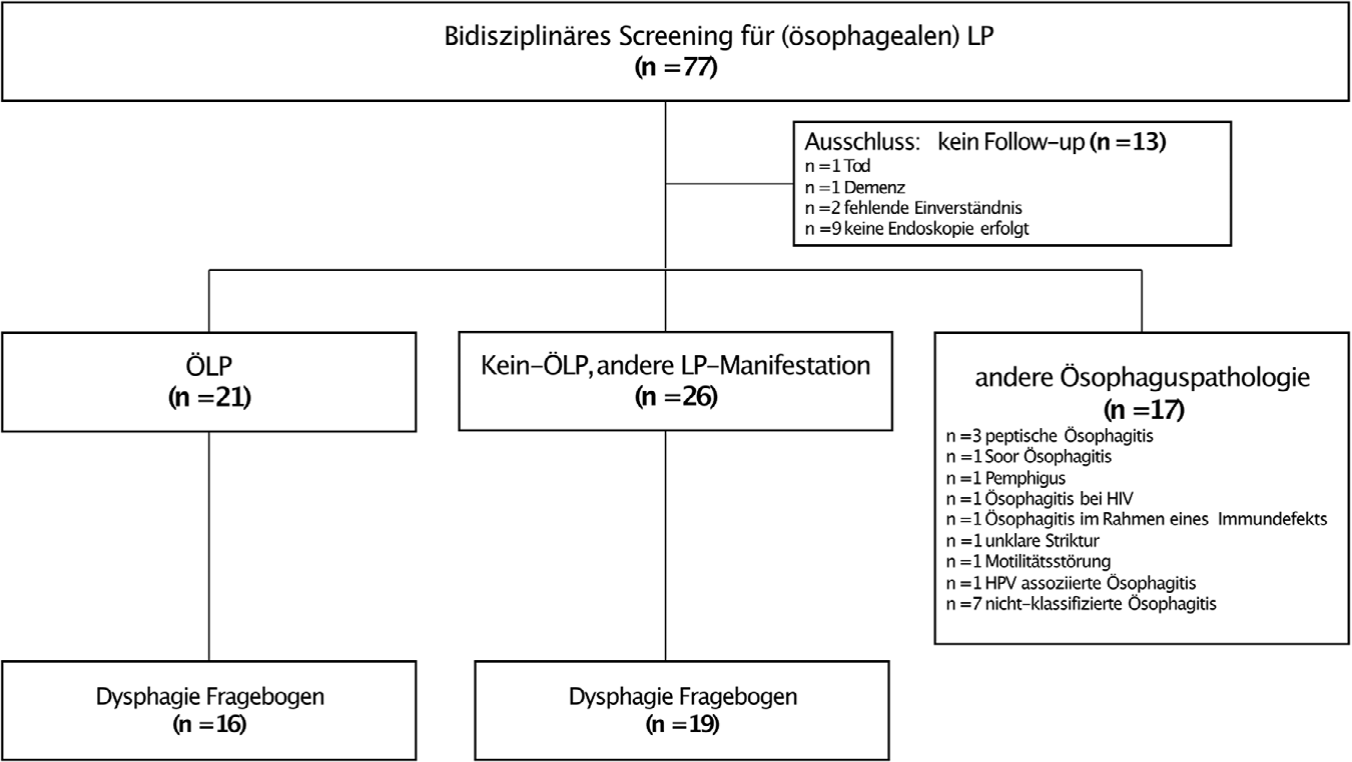
Patientenrekrutierung: Insgesamt wurden 77 Patienten in die Studie eingeschlossen. Einbezogen wurden Patienten, die von der Gastroenterologie mit Verdacht auf ÖLP zur dermatologischen Mitbeurteilung überwiesen wurden, sowie Patienten aus der Dermatologie mit bestätigtem LP an beliebiger Lokalisation, die über Dysphagie berichteten und daher eine endoskopische Untersuchung benötigten. Dreizehn Patienten wurden aufgrund eines fehlenden Follow‐up ausgeschlossen, und bei 17 Patienten wurden andere Ösophaguspathologien diagnostiziert. Von den verbleibenden 47 Patienten erhielten 21 die Diagnose ÖLP, während 26 LP an anderen Lokalisationen, jedoch ohne Ösophagusbeteiligung aufwiesen.

**ABBILDUNG 2:**
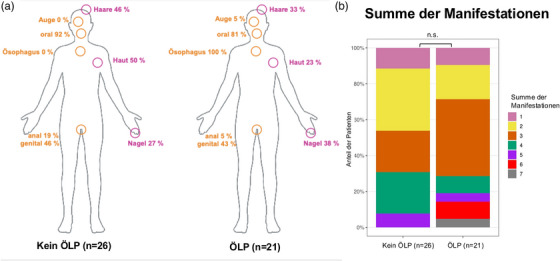
(a) Verteilung der LP‐Begleitmanifestationen bei Patienten ohne ÖLP (n = 26) und mit ÖLP (n = 21). Haut‐ und Analbeteiligungen traten in der ÖLP‐Gruppe im Vergleich zur Nicht‐ÖLP‐Gruppe zwar seltener auf, diese Unterschiede waren jedoch nicht statistisch signifikant. (b) Vergleich der Gesamtzahl der LP‐Begleitmanifestationen zwischen Nicht‐ÖLP‐Gruppe (n = 26) und ÖLP‐Gruppe (n = 21). Obwohl kein signifikanter Unterschied zwischen den Gruppen beobachtet wurde (p > 0,05), befanden sich auffälligerweise alle drei Patienten mit der höchsten Anzahl an Manifestationen (6 oder 7) in der ÖLP‐Gruppe.*Abk*.: n.s. significant

Wir untersuchten 562 Patienten auf symptomatischen und klinisch relevanten ÖLP. Durch diesen Ansatz wurden 77 Patienten (n = 50 aus der Dermatologie, n = 27 aus der Gastroenterologie) mit Verdacht auf potenziellem ÖLP eingeschlossen (Abbildung 1). Dreizehn Patienten wurden aufgrund von Demenz, Tod oder Rücknahme der Einwilligung zur Endoskopie ausgeschlossen. Bei 17 der verbliebenen 64 Patienten wurden durch die endoskopischen, histopathologischen und dermatologischen Untersuchungen keine LP‐Manifestationen gefunden und wurde für die weitere Analyse nicht berücksichtigt. Bei 21 Patienten wurde ein ÖLP diagnostiziert (ÖLP‐Gruppe), während 26 Patienten Dysphagie ohne bestätigte Ösophaguspathologie, aber einen LP in anderen Bereichen aufwiesen (Nicht‐ÖLP‐Gruppe) (Abbildung 2). Die abschließende Analyse umfasste daher 47 Patienten mit einer bestätigten (Ö)LP‐Diagnose basierend auf der dermatologischen und endoskopischen Beurteilung.

Alle ÖLP‐Fälle erfüllten die veröffentlichten vollständigen diagnostischen Kriterien^20^ (Online‐Supplement Tabelle S1, Nr. 1–21). Zwei Patienten wiesen basierend auf diesen Kriterien eine schwere Form auf (Online‐Supplement Tabelle S1, Nr. 7–8), und zwei Patienten hatten einen isolierten ÖLP (Online‐Supplement Tabelle S1, Nr. 17,18). Bei insgesamt 562 LP‐Patienten, die während des Studienzeitraums in unserem Zentrum behandelt wurden, identifizierten wir in 3,7% der Fälle einen symptomatischen ÖLP.

Das mediane Alter in der ÖLP‐Gruppe betrug 72 Jahre, verglichen mit 57 Jahren in der Nicht‐ÖLP‐Gruppe. Frauen waren in beiden Gruppen häufiger betroffen und machten 71% der ÖLP‐Gruppe und 88% der Nicht‐ÖLP‐Gruppe aus. Der mediane Body‐Mass‐Index (BMI) war in der ÖLP‐Gruppe (24 kg/m^2^) niedriger als in der Nicht‐ÖLP‐Gruppe (26 kg/m^2^). Die Raucherquoten waren in beiden Gruppen ähnlich, mit 33% in der ÖLP‐Gruppe und 38% in der Nicht‐ÖLP‐Gruppe (Tabelle 2).

**TABELLE 2:** Patientencharakteristika.

	ÖLP (n = 21)	Kein ÖLP (n = 26)
Durchschnittsalter [Jahre]	72 [q1: 65; q3: 80]	57 [q1: 46; q3: 67]
Frauen [%]	71% (15/21)	88% (23/26)
Mittlerer BMI [kg/m^2^]	24 [q1: 22; q3: 26] (9 NA)	26 [q1: 22; q3: 29] (14 NA)
Rauchen	33% (5/15, 6 NA)	38% (6/16, 10 NA)
Hepatitis‐C‐Virus‐Infektion	5% (1/20)	4% (1/26)
Hepatitis‐B‐Virus‐Infektion	5% (1/20)	8% (2/26)
Ösophageales PEK	10% (2/20)	0% (0/26)

*Abk*.: BMI, Body‐Mass‐Index; ÖLP, Ösophagus‐Lichen‐planus; NA, nicht verfügbar; q1, erstes Quartil; q3, drittes Quartil; PEK, Plattenepithelkarzinom

Eine Vorgeschichte von HCV‐Infektion war bei 5% der ÖLP‐Gruppe und 4% der Nicht‐ÖLP‐Gruppe vorhanden. In der ÖLP‐Gruppe bezog sich dies auf Patienten, die zuvor erfolgreich wegen einer HCV‐Infektion behandelt worden waren, während in der Nicht‐ÖLP‐Gruppe bei einem Patienten aufgrund unseres Screenings eine HCV‐Infektion neu diagnostiziert wurde. Bezüglich der HBV‐Infektion gab es in jeder Gruppe einen Fall von chronischer HBV‐Infektion sowie einen Fall von ausgeheilter HBV‐Infektion in der Nicht‐ÖLP‐Gruppe. Bemerkenswert ist, dass bei zwei der 21 Patienten in der ÖLP‐Gruppe ein PEK der Speiseröhre im UICC‐Stadium I diagnostiziert wurde, während in der Nicht‐ÖLP‐Gruppe keine Fälle von PEK gefunden wurden (Tabelle 2).

### Endoskopische und histologische Befunde

Während der Endoskopie wurde bei 62% der Patienten eine Mukosadenudation, ein spezifischer makroskopischer Befund, beobachtet. Die Mehrheit der Fälle wurde als D1 (52%) klassifiziert, während nur zwei Patienten (10%) als D2 eingestuft wurden, was gemäß dem Graduierungssystem als schwerer ÖLP gilt.^20^ Unspezifische endoskopische Befunde waren häufig und traten bei 81% der Patienten auf. Bei den Patienten wurden Hyperkeratosen in 42% der Fälle und eine Trachealisierung in 71% der Fälle festgestellt. Eine endoskopisch passierbare Stenose (S1) wurde bei 29% der Patienten beobachtet, während 33% eine S2‐Stenose hatten, und 29% benötigten eine endoskopische Dilatation. Bei 19% der Patienten wurde eine Ösophagus‐Candidose diagnostiziert, wahrscheinlich als Nebenwirkung der topischen Steroidbehandlung. Charakteristische histologische ÖLP‐Befunde wurden bei 90% der Patienten festgestellt. In 92% der Fälle, bei denen eine DIF durchgeführt wurde, zeigten diese Fibrinogenablagerung (Tabelle 3).

**TABELLE 3:** Endoskopische, histologische und Immunfluoreszenzbefunde in der ÖLP‐Gruppe.

Endoskopische Kriterien ÖLP (n = 21)			
Denudation		13/21 (62%)	
	D1		11 (52%)
	D2		2 (10%)
	D3		0 (0%)
Unspezifische Kriterien		17/21 (81%)	
	Hyperkeratose		9 (42%)
	Trachealisierung		15 (71%)
	Stenose		13/21 (62%)
	S1		6 (29%)
	S2		7 (33%)
Histologische Kriterien ÖLP (n = 21)			
*Charakteristische ÖLP‐Histologie ≥ HP1*		*19/21 (90%)*	
	Civatte‐Körperchen		7 (33%)
	Dyskeratose		12 (57%)
	Epithelablösung		7 (33%)
	Lymphozytäres Infiltrat		16 (76%)
Direkte Immunfluoreszenz (n = 14, 7 NA)			
Positive Fibrinogenablagerung in DIF ≥ F1		13/14 (92%)	
	F1 (schwach)		5 (35%)
	F2 (stark)		8 (57%)

*Abk*.: DIF, direkte Immunfluoreszenz; ÖLP, Ösophagus‐Lichen‐planus; HP, Histopathologie; NA, nicht verfügbar; PEK, Plattenepithelkarzinom; S, Stenose

Die häufigste zusätzliche klinische LP‐Manifestationsstelle in beiden Gruppen war der OLP (81% ÖLP‐Gruppe, 92% Nicht‐ÖLP‐Gruppe) (Abbildung 2a). Eine Haut‐ und Analbeteiligung war bei einem ÖLP weniger häufig als bei Nicht‐ÖLP‐Fällen, obwohl die Unterschiede nicht statistisch signifikant waren. Andere Manifestationen (Nagel, Haar, Genital) traten in beiden Gruppen mit ähnlicher Häufigkeit auf. Es gab keinen statistisch signifikanten Unterschied in der Gesamtzahl der Manifestationen zwischen der ÖLP‐ und Nicht‐ÖLP‐Gruppe. Bemerkenswert ist jedoch, dass bei den drei Patienten mit der höchsten Anzahl von LP‐Manifestationen (6 oder 7) ein ÖLP diagnostiziert wurde (Abbildung 2b). Auf Grund der kleinen Stichprobengröße können daraus jedoch keine robusten Schlussfolgerungen gezogen werden. Andererseits hatten zwei ÖLP‐Patienten keine weiteren LP‐Manifestation, wiesen also einen isolierten Befall der Speiseröhre auf.

### Befunde des Dysphagie‐Fragebogens

**ABBILDUNG 3:**
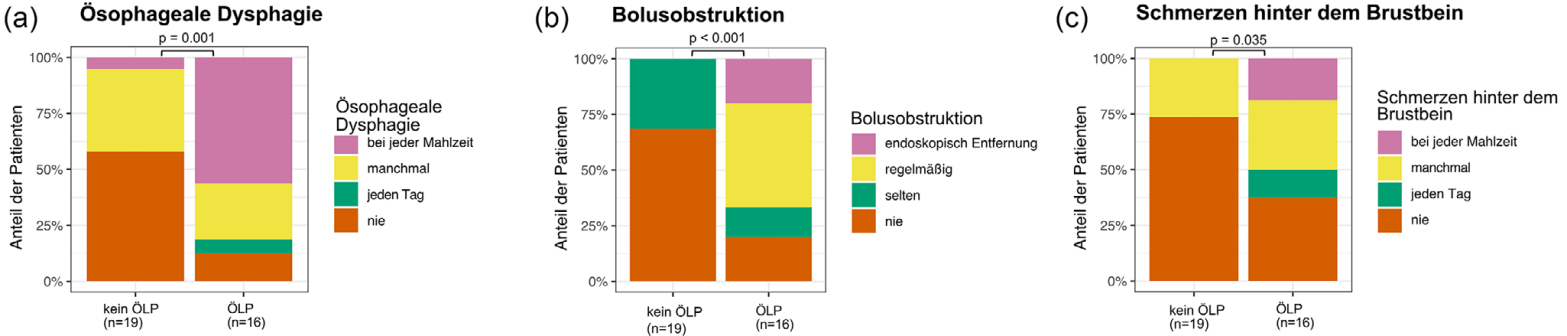
Klinische Fragen, die signifikant mit ÖLP korrelieren: (a) ösophageale Dysphagie: (b) Bolusobstruktion; (c) retrosternale Schmerzen.

Die Dysphagie‐Fragebögen wurden von 19 Patienten in der Nicht‐ÖLP‐Gruppe und von 16 Patienten in der ÖLP‐Gruppe ausgefüllt. Signifikante Unterschiede (p < 0,05) wurden zwischen den beiden Gruppen bei drei Fragen gefunden: *(1) Ösophageale Dysphagie*: 56% der ÖLP‐Patienten berichteten über Schluckbeschwerden bei jeder Mahlzeit, verglichen mit nur 5% derjenigen ohne ÖLP (Abbildung 3a). *(2) Bolusobstruktion*: In der ÖLP‐Gruppe berichteten 44% der Patienten über regelmäßiges Steckenbleiben von Nahrung, und 19% hatten in der Vergangenheit mindestens einmal eine endoskopische Entfernung eines Nahrungsbolus benötigt. Im Gegensatz dazu berichtete keiner der Patienten ohne ÖLP über Steckenbleiben von Nahrung oder benötigte eine endoskopische Bolusentfernung (Abbildung 3b). *(3) Retrosternale Schmerzen*: 19% der ÖLP‐Patienten erlebten retrosternale Schmerzen bei jeder Mahlzeit, ein Symptom, das von keinem der Patienten in der Nicht‐ÖLP‐Gruppe berichtet wurde (Abbildung 3c).

## DISKUSSION

Wir präsentieren Daten aus einer prospektiven Kohortenstudie zum Screening auf klinisch relevanten ÖLP unter Verwendung eines bidisziplinären Ansatzes. Für die Diagnose des ÖLP wandten wir die zuvor veröffentlichten Kriterien an, die endoskopische, histologische und DIF‐Beurteilungen umfassen.^20^ Mit diesem Ansatz identifizierten wir 21 LP‐Patienten mit ÖLP und 26 Patienten ohne ösophageale Manifestation (beide mit Dysphagie) und konnten klinische Details der beiden Gruppen vergleichen. Als Ergänzung zu unseren früheren Daten korrelierte die klinische Schwere bei ÖLP nicht unbedingt streng mit dem Ausmaß der Mukosadenudation, einem typischen Kriterium, das mittels Endoskopie objektiviert werden kann.^20^ Stattdessen schien die Schwere zusätzliche Faktoren wie den Grad der histologischen Entzündung, klinische Symptome und Komplikationen wie Stenosen zu umfassen. Daher schlagen wir vor, die bisherige Unterteilung in „milde“ und „schwere“ ÖLP‐Formen anhand der Mukosadenudation nicht weiter zu verwenden, da sie die therapeutische Entscheidungsfindung eher erschwert.

Ösophageale Dysphagie kann ein Symptom verschiedener Erkrankungen sein, darunter Malignome, peptische Strikturen aufgrund von Refluxösophagitis, Infektionen, Motilitätsstörungen und entzündliche Ursachen.^16^, ^18^, ^27^ Die Diagnose des ÖLP ist aufgrund seiner symptomatischen Ähnlichkeit mit diesen Erkrankungen eine Herausforderung. Die EoE kann histologisch klar vom ÖLP unterschieden werden, wobei eine EoE typischerweise durch mehr als 15 Eosinophile pro *High Power Field* gekennzeichnet ist.^28^ Eine GERD kann von einem ÖLP über eine Endoskopie oder in unklaren Fällen durch eine Kombination aus Endoskopie und Histologie unterschieden werden.^23^ Einige Experten betrachten ÖLP und lichenoide Ösophagitis als Varianten der lymphozytären Ösophagitis, obwohl der ÖLP histologisch durch apoptotische Keratinozyten, die als Civatte‐Körperchen bezeichnet werden, gekennzeichnet ist, die typischerweise bei der lichenoiden Ösophagitis fehlen.^16^, ^27^, ^29^ Obwohl eine lichenoide Ösophagitis klinisch einem ÖLP ähneln kann, ist Letzterer häufiger mit der Entwicklung von Ösophagusstenosen verbunden.^27^ Wir stellten fest, dass für die Diagnose eines ÖLP entscheidend ist, dass diese von mit der Erkrankung vertrauten Ärzten durchgeführt wird. Auch wenn die histologische Auswertung in manchen Fällen nicht zur Diagnose beitrug, verbesserte die Überprüfung durch einen erfahrenen Pathologen die diagnostische Genauigkeit. Um histologische Befunde beurteilen zu können, die mit einem ÖLP übereinstimmen, benötigen Pathologen Proben, die Ösophagusstroma und nicht nur abgelöstes Epithel enthalten. Die Sicherstellung einer ausreichenden Biopsietiefe ist daher in der klinischen Praxis entscheidend. In unserer Kohorte war dies kein Problem, vermutlich dank der Erfahrung der Untersucher, was die Bedeutung einer gezielten Schulung von Klinikern unterstreicht. Nach unserer Erfahrung steigt die diagnostische Genauigkeit, wenn alle diagnostischen Komponenten – Makroskopie, Histologie und DIF – berücksichtigt werden. In unserer Kohorte war die externe Diagnostik teils unvollständig – insbesondere wurde die DIF‐Mikroskopie extern nie berücksichtigt. Da die diagnostischen Kriterien für ÖLP bei Gastroenterologen und Pathologen möglicherweise nicht ausreichend bekannt sind, kann dies zu falsch‐negativen Befunden führen, wodurch die Prävalenz der Erkrankung unterschätzt werden könnte. Dies macht den dringenden Bedarf an Wissenstransfer über den ÖLP innerhalb der ärztlichen Fachgruppen deutlich. Die Diagnose kann durch interdisziplinäre Zusammenarbeit von geschulten Gastroenterologen, Dermatologen und Pathologen gestellt werden.

In unserer Kohorte waren Patienten mit diagnostiziertem ÖLP in der Regel älter als diejenigen ohne ÖLP, und Frauen waren mit 71% der Fälle häufiger betroffen, was mit früheren Berichten in der Literatur übereinstimmt.^30^ Patienten in der Nicht‐ÖLP‐Gruppe hatten am häufigsten eine orale Beteiligung, was darauf hindeutet, dass ihre berichtete Dysphagie eher oropharyngealer als ösophagealer Natur war. Es gab keinen signifikanten Unterschied der Manifestationsstellen zwischen den Gruppen, jedoch traten Haut‐ und Analbeteiligungen bei ÖLP‐Patienten tendenziell seltener auf. Die Anzahl der LP‐Manifestationen unterschied sich nicht zwischen den beiden Gruppen, jedoch wurden alle Patienten mit der höchsten Anzahl von Manifestationen (6 oder mehr) mit ÖLP diagnostiziert, was darauf hindeutet, dass Personen mit multilokulärem LP ein höheres Risiko haben könnten, einen ÖLP zu entwickeln. Dies unterstreicht die Bedeutung eines interdisziplinären Ansatzes bei der Diagnose und Behandlung von (Ö)LP, wobei zu betonen ist, dass die Wahrscheinlichkeit einer ösophagealen Beteiligung bei Patienten mit weiteren LP‐Manifestationen höher ist. Dies verdeutlicht die Notwendigkeit einer dermatologischen Untersuchung, um falsch negative Interpretationen endoskopischer und histologischer Befunde zu vermeiden.^20^ Zwei Patienten hatten einen isolierten ÖLP ohne mukokutane Manifestationen, wie auch in der Literatur bekannt.^20^

Basierend auf oben genannten Überlegungen und unserer Erfahrung bei der Anwendung der bisherigen Kriterien^20^ schlagen wir einen überarbeiteten und vereinfachten Ansatz für die Diagnose des ÖLP vor. Die Diagnose könnte in folgenden Szenarien gestellt werden: *(1)* Endoskopische Denudation (D) jeden Grades, begleitet entweder von einem positiven histopathologischen Befund oder positiver DIF für Fibrinogenablagerungen. *(2)* Mindestens ein endoskopisches Zeichen (Stenose, Hyperkeratose, Trachealisierung) zusammen mit entweder einem positiven histopathologischen Ergebnis oder einem positiven DIF‐Ergebnis, oder zusammen mit dermatologischer Bestätigung der LP‐Manifestation in anderen Bereichen und histologischem Ausschluss anderer häufiger ösophagealer Differenzialdiagnosen (Online‐Supplement Tabelle S2).

Bei zwei Patienten in der ÖLP‐Gruppe (10%) wurde ein ösophageales PEK diagnostiziert, was die Einstufung als fakultative Präkanzerose analog zum OLP unterstreicht. In der Literatur wird das PEK‐Risiko mit 5–6% angegeben.^18^, ^31^ Daraus folgt die Notwendigkeit einer endoskopischen Nachsorge bei Patienten mit bekanntem ÖLP, um eine potenzielle Transformation in ein PEK frühzeitig zu erfassen.^18^, ^31^ Einer unserer Patienten hatte in der Vorgeschichte eine chronischen HCV‐Infektion, die mehrere Jahre vor der Diagnose eines PEK mit antiviraler Behandlung geheilt worden war. Die Infektion diente möglicherweise als Auslöser für den ÖLP, während die daraus resultierende persistierende chronische Entzündung zur Entwicklung des PEK beigetragen haben könnte. Es gab jedoch keinen signifikanten Unterschied in der Prävalenz von HBV‐ und HCV‐Infektionen zwischen den ÖLP‐ und Nicht‐ÖLP‐Gruppen.

Von den 64 Patienten, die in unserer Studie aufgrund des Verdachts auf ÖLP interdisziplinär untersucht wurden, konnten bei 17 Patienten letztlich keine LP‐Manifestationen festgestellt werden. Dies ist auf das Studiendesign mit Screening gastroenterologischer Patienten mit hoher Wahrscheinlichkeit für ÖLP zurückzuführen. Von den verbleibenden 47 Patienten, die alle einen bestätigten LP an einer oder mehreren Stellen hatten und ösophageale Symptome aufwiesen, wurden 44,6% (21/47) mit ÖLP diagnostiziert. Wenn man die Gesamtzahl von 562 Patienten betrachtet, die in der vierjährigen Studienperiode an unserem Zentrum wegen einer diagnostizierten LP‐Manifestation behandelt wurden, diagnostizierten wir ÖLP bei 3,7% (21/562) der Fälle unter Verwendung des symptombasierten Screening‐Ansatzes. Dies könnte aufgrund des Ausschlusses von 13 symptomatischen Patienten, die im Follow‐up verloren gingen unterschätzt sein. Ob dies eine wahre Prävalenz darstellt, bleibt wegen der vorausgewählten Patientenpopulation spekulativ. Angesichts des Studiendesigns müssen unsere Schlussfolgerungen mit Vorsicht interpretiert werden. Eine prospektive, multizentrische Studie mit systematischem Screening asymptomatischer Patienten ist erforderlich, um die tatsächliche Prävalenz zu bestimmen und die Krankheitscharakterisierung weiter zu verfeinern. Dennoch zeigen diese Ergebnisse, dass ein klinisch manifester ÖLP in der Allgemeinbevölkerung zwar eine seltene Erkrankung darstellt, bei bis zu 45% der Patienten mit bekanntem LP und Dysphagie jedoch eine Ösophagusbeteiligung diagnostiziert wurde. Ein endoskopisches Screening dieser vorselektierten Gruppe sollte daher in der klinischen Praxis empfohlen werden. Wir konnten zeigen, dass Patienten mit ÖLP drei unterschiedliche Symptome im Vergleich zur Nicht‐ÖLP‐Gruppe aufwiesen: *(1)* häufige ösophageale Dysphagie mit Schluckproblemen bei jeder Mahlzeit; *(2)* häufige Nahrungsmittelbolusereignisse, die in einigen Fällen eine endoskopische Entfernung erforderte; *(3)* häufige retrosternale Schmerzen. Die Ergebnisse zeigen, dass ein gezielter Fragebogen, der sich auf diese spezifischen Symptome konzentriert, als Screening‐Tool für Dermatologen dienen könnte, um diejenigen LP‐Patienten zu erkennen, die ein endoskopisches Screening auf ÖLP benötigen.

Aufgrund der Vorselektion und des Designs der aktuellen Studie könnten wir jedoch Patienten mit asymptomatischem ÖLP übersehen haben.^21^, ^22^ Obwohl relativ selten, wurden asymptomatische Fälle beschrieben.^19^, ^20^ Fox et al. beschreiben beispielsweise, dass 17% von 72 Patienten mit ÖLP entweder asymptomatisch waren oder lediglich minimale Symptome zeigten.^18^ Zukünftige Studien sollten diese Faktoren berücksichtigen und asymptomatische Fälle einschließen. Während die Diagnose eines ÖLP bei asymptomatischen Patienten in erster Linie akademischen Zwecken dient, sollten Behandlungsentscheidungen auf der Grundlage klinischer Symptome sowie endoskopischer und histologischer Befunde getroffen werden.^23^ Dennoch kann die Identifizierung solcher Fälle entscheidend sein, da ein potenziell erhöhtes Risiko für die Entwicklung eines ösophagealen PEK besteht. Aus wirtschaftlichen Gründen und aufgrund des Komplikationsrisikos ist es jedoch nicht praktikabel, regelmäßige Endoskopien zum Zweck des Tumorscreenings bei allen Patienten mit LP durchzuführen, wenn keine ösophagealen Symptome vorliegen. Aus denselben Gründen ist das Screening auf Speiseröhrenkrebs für die Allgemeinbevölkerung in Ländern mit niedriger Prävalenz, wie Deutschland, nicht etabliert.^32^ Wir schlagen daher vor, dass ein gezielter Fragebogen, der die wichtigsten ÖLP‐Symptomen abfragt (siehe oben), als effektives Screening‐Tool für LP‐Patienten dienen könnte. Auf diese Weise ließe sich ein günstigeres Verhältnis zwischen Nutzen und Risiko einer Endoskopie erreichen.

Wir empfehlen die Behandlung eines ÖLP bei Vorliegen von Dysphagie, endoskopischen oder histologischen Anzeichen einer Entzündung und/oder einer Stenose. Das Hauptziel ist die Linderung der Symptome, die Behandlung von Stenosen und die Verhinderung anderer Langzeitkomplikationen (PEK‐Risiko). Ein besonderes Merkmal des ÖLP ist, dass sich Stenosen unter antiinflammatorischer Therapie zurückbilden können, sodass eine Dilatation oft nicht erforderlich ist.^20^ In unserem Zentrum hat sich topisches Budesonid als die wirksamste Behandlung erwiesen, entweder als viskose Lösung oder als orodispersible Tabletten, die ursprünglich für die EoE entwickelt wurden (Online‐Supplement Tabelle S3).^20^, ^33^ Darüber hinaus werden derzeit T‐Zell‐wirksame Therapien, Biologika und *small molecules* in der ÖLP‐Behandlung eingesetzt.^3^ JAK‐Inhibitoren stellen die vielversprechendste therapeutische Alternative dar.^34^, ^35^ Zu beachten ist, dass sich alle diese Behandlungsoptionen derzeit außerhalb der Zulassung befinden.^36^

## DANKSAGUNG

R.D. erhielt beratende bei der Datenanalyse Unterstützung vom *Institut für Medizinische Biometrie und Statistik (IMBI)* in Freiburg, insbesondere zur Sicherstellung der Anwendung geeigneter statistischer Tests.

Open access Veröffentlichung ermöglicht und organisiert durch Projekt DEAL.

## FINANZIERUNG

R.D. wird gefördert durch die Deutsche Forschungsgemeinschaft (DFG) – CRC1160/2 – B03(N), das Universitätsklinikum Freiburg, die Medizinische Fakultät der Albert‐Ludwigs‐Universität Freiburg sowie durch das Berta‐Ottenstein‐Programm für *Clinician Scientists* der Medizinischen Fakultät der Albert‐Ludwigs‐Universität Freiburg. F.S. wurde durch das Berta‐Ottenstein‐Programm *Advanced Clinician Scientist* der Medizinischen Fakultät der Albert‐Ludwigs‐Universität Freiburg gefördert.

## INTERESSENKONFLIKT

Keiner.

## Supporting information



Supplementary information
