# Supplementary material for: Lichen planus des Ösophagus: Eine prospektive, interdisziplinäre, monozentrische Kohortenstudie
Source: J Dtsch Dermatol Ges. 2025 Nov 14;23(11):1394–402. [Article in German] doi: 10.1111/ddg.15808_g (PMC12619047; doi:10.1111/ddg.15808_g)
Supplement: Supplementary file 1 — Supplementary information [file DDG-23-1394-s001.docx]

[[online supplement]]

Online-Supplement TABELLE S1: Endoskopische, histologische, immunfluoreszenz und klinische Charakteristika der ÖLP Patienten.

| **Nr.** | **Alter** | **Sex** | **H** | **T** | **D1** | **D2** | **D3** | **S1** | **S2** | **Dilatation** | **Soor** | **HP** | **Civatte-Körperchen** | **Dyskeratose** | **Ablösung des Epithels** | **Lymphozytäres Infiltrat** | **F0** | **F1 (schwach)** | **F2 (stark)** | **Endoskopisch + Histologisch + DIF** | **Oral** | **Genital** | **Anal** | **Haut** | **Haar** | **Nagel** | **Auge** |
| --- | --- | --- | --- | --- | --- | --- | --- | --- | --- | --- | --- | --- | --- | --- | --- | --- | --- | --- | --- | --- | --- | --- | --- | --- | --- | --- | --- |
| 1 | 85 | M | Nein | Ja | Ja | Nein | Nein | Nein | Ja | Ja | Nein | HP3 | Nein | Ja | Ja | Ja | Nein | Nein | Ja | T, D1, S2, Dilation, HP3, F2 | Ja | Nein | Nein | Nein | Nein | Nein | Nein |
| 2 | 63 | F | Nein | Ja | Nein | Nein | Nein | Nein | Ja | Nein | Ja | HP3 | Nein | Ja | Ja | Ja | Nein | Ja | Nein | T, S2, HP3, F1 | Ja | Ja | Nein | Ja | Ja | Ja | Nein |
| 3 | 68 | F | Ja | Ja | Ja | Nein | Nein | Ja | Nein | Nein | Ja | HP3 | Ja | Nein | Ja | Ja | Nein | Nein | Ja | H, T, D1, S1, HP3, F1 | Ja | Nein | Nein | Nein | Ja | Nein | Nein |
| 4 | 75 | M | Nein | Ja | Nein | Nein | Nein | Nein | Ja | Nein | Nein | HP2 | Ja | Nein | Nein | Ja | Nein | Nein | Ja | T, S2, HP3, F2 | Ja | Nein | Nein | Nein | Ja | Nein | Nein |
| 5 | 80 | M | Nein | Ja | Ja | Nein | Nein | Nein | Ja | Ja | Nein | HP3 | Nein | Ja | Ja | Ja | Ja | Nein | Nein | T, D1, S1, Dilation, HP3, F0 | Ja | Nein | Nein | Nein | Ja | Ja | Nein |
| 6 | 70 | F | Nein | Nein | Nein | Nein | Nein | Ja | Nein | Nein | Ja | HP3 | Nein | Ja | Ja | Ja | Nein | Nein | Ja | S1, HP3, F2 | Ja | Nein | Nein | Nein | Nein | Ja | Nein |
| 7 | 66 | F | Ja | Ja | Nein | Ja | Nein | Nein | Ja | Ja | Nein | HP2 | Ja | Ja | Nein | Nein | Nein | Ja | Nein | H, T, D2, S2, Dilation, HP2, F1 | Ja | Ja | Nein | Ja | Ja | Nein | Nein |
| 8 | 79 | F | Ja | Ja | Nein | Ja | Nein | Ja | Nein | Ja | Nein | HP2 | Nein | Ja | Nein | Ja | NA | NA | NA | H, T, D2, S1, Dilation, HP2, FNA | Ja | Ja | Ja | Ja | Nein | Ja | Nein |
| 9 | 83 | F | Nein | Nein | Ja | Nein | Nein | Nein | Nein | Nein | Nein | HP1 | Nein | Nein | Nein | Ja | NA | NA | NA | D1, HP1, FNA | Ja | Nein | Nein | Nein | Nein | Ja | Nein |
| 10 | 74 | F | Ja | Ja | Nein | Nein | Nein | Nein | Nein | Nein | Nein | HP1 | Nein | Nein | Nein | Ja | Nein | Nein | Ja | H, T, HP1, F2 | Ja | Ja | Nein | Nein | Nein | Nein | Nein |
| 11 | 56 | F | Ja | Ja | Ja | Nein | Nein | Nein | Nein | Nein | Nein | HP0 | Nein | Nein | Nein | Nein | Nein | Nein | Ja | H, T, D1, HP0, F2 | Ja | Nein | Nein | Ja | Nein | Nein | Nein |
| 12 | 63 | F | Nein | Nein | Ja | Nein | Nein | Nein | Nein | Nein | Nein | HP0 | Nein | Nein | Nein | Nein | Nein | Ja | Nein | D1, HP0, F1 | Ja, Zahnfleischretraktion | Ja | Nein | Nein | Nein | Nein | Nein |
| 13 | 87 | M | Nein | Nein | Nein | Nein | Nein | Nein | Nein | Nein | Nein | HP1 | Nein | Nein | Nein | Ja | Nein | Nein | Ja | HP1, F2 | Ja | Nein | Nein | Nein | Nein | Nein | Nein |
| 14 | 65 | F | Nein | Nein | Ja | Nein | Nein | Nein | Nein | Nein | Nein | HP2 | Ja | Nein | Nein | Ja | Nein | Nein | Ja | D1, HP2, F2 | Ja | Ja | Nein | Nein | Nein | Nein | Nein |
| 15 | 87 | F | Ja | Ja | Nein | Nein | Nein | Ja | Nein | Ja | Nein | HP2 | Nein | Ja | Nein | Ja | NA | NA | NA | H, T, S1, HP2. FNA | Ja | Nein | Nein | Nein | Nein | Ja | Nein |
| 16 | 75 | F | Ja | Ja | Ja | Nein | Nein | Nein | Nein | Nein | Nein | HP3 | Ja | Ja | Ja | Nein | NA | NA | NA | H, T, D1, HP3, FNA | Ja | Ja | Nein | Nein | Nein | Nein | Nein |
| 17 | 75 | F | Nein | Ja | Ja | Nein | Nein | Nein | Ja | Nein | Nein | HP2 | Ja | Nein | Nein | Ja | Nein | Ja | Nein | T, D1, S2, HP2, F1 | Nein | Nein | Nein | Nein | Nein | Nein | Nein |
| 18 | 62 | F | Ja | Ja | Ja | Nein | Nein | Ja | Nein | Nein | Ja | HP3 | Ja | Ja | Ja | Ja | Nein | Ja | Nein | H, T, D1, S1, HP3, F1 | Nein | Nein | Nein | Nein | Nein | Nein | Nein |
| 19 | 79 | M | Ja | Ja | Nein | Nein | Nein | Nein | Nein | Nein | Nein | HP2 | Nein | Ja | Nein | Ja | NA | NA | NA | H, T, HP2, FNA | Ja, Zahnfleischretraktion | Nein | Nein | Nein | Nein | Ja | Nein |
| 20 | 45 | F | Nein | Ja | Nein | Nein | Nein | Nein | Ja | Ja | Nein | HP1 | Nein | Ja | Nein | Nein | NA | NA | NA | T, S2, Dilation, HP1, FNA | Ja | Ja | Nein | Ja | Nein | Ja | Ja |
| 21 | 81 | F | Nein | Nein | Ja | Nein | Nein | Ja | Nein | Nein | no | HP1 | Nein | Nein | Ja | Ja | NA | NA | NA | D1, S1, HP1 | Ja | Ja | Nein | Nein | Ja | Nein | Nein |

*Abk.:* D, Denudation; DIF, direkte Immunfluoreszenz; F, Fibrinogenablagerung in der DIF; H, Hyperkeratose; HP, Histopathologie; NA, nicht verfügbar; S, Stenose; SCC, Plattenepithelkarzinom; T, Trachealisierung

Online-Supplement TABELLE S2: Das adaptierte erste Kriterium fasst die beiden bisherigen Kriterien 1 und 2 zusammen und entfernt die frühere Klassifizierung in „milde“ und „schwere“ Formen basierend auf dem Ausmaß der Schleimhautablösung, um die Diagnosestellung zu vereinfachen. Das bisherige Kriterium 3 wurde erweitert, um die Bedeutung einer umfassenden dermatologischen Untersuchung hervorzuheben. Damit wird anerkannt, dass andere LP-Manifestationen wertvolle diagnostische Hinweise geben können, insbesondere in Fällen mit subtilen oder unspezifischen endoskopischen und histologischen Befunden.

| Alte diagnostische Kriterien | Adaptierte diagnostische Kriterien |
| --- | --- |
| 1. ≥ D2 und HP ≥ 1 und/oder F ≥ 1, entspricht schwerem ÖLP 2. D1 und HP ≥ 1 und/oder F ≥ 1 3. S, H, T oder jeder Fall von keinen oder unklaren endoskopischen Befunden, aber but HP ≥ 1 und F ≥ 1 | 1. ≥ D1 und HP ≥ 1 und/oder F ≥ 1 2. S, H, T und ein weiteres Kriterium:  - HP ≥ 1 oder - F ≥ 1 oder - Dermatologische LP-Manifestation an anderen Stellen und histologischer Ausschluss relevanter Differenzialdiagnosen |

*Abk.:* D, Denudation; DIF, direkte Immunfluoreszenz; F, Fibrinogenablagerung in der DIF; H, Hyperkeratose; HP, Histopathologie; LP, Lichen planus; S, Stenose; T, Trachealisierung

Online Supplement TABELLE S3: ÖLP-Patienten: Therapien und Therapieansprechen.

| **Nr.** | **Alter** | **Geschlecht** | **Endoskopische + Histologisch + DIF** | **Therapie** | **Outcome** |
| --- | --- | --- | --- | --- | --- |
| 1 | 85 | M | T, D1, S2, Dilation, HP3, F2 | Topisches Budesonid | Reduzierte Entzündungsreaktion, Verbesserung der S |
| 2 | 63 | W | T, S2, HP3, F1 | Topisches Budesonid, orodispergierbare Tabletten | Reduzierte Entzündungsreaktion, Verbesserung der S |
| 3 | 68 | W | H, T, D1, S1, HP3, F1 | Topisches Budesonid | Komplette Remission von S und T |
| 4 | 75 | M | T, S2, HP3, F2 | Topisches Budesonid | Keine Reendoskopie in unserem Zentrum |
| 5 | 80 | M | T, D1, S1, Dilation, HP3, F0 | Topisches Budesonid | Reduzierte Entzündungsreaktion, wiederkehrende Pilz-Ösophagitis, Verbesserung von S |
| 6 | 70 | W | S1, HP3, F2 | Topisches Budesonid | Keine Reendoskopie in unserem Zentrum |
| 7 | 66 | W | H, T, D2, S2, Dilation, HP2, F1 | Topisches Budesonid, Mycophenolatmofetil, Cyclophosphamid, Tofacitinib | Keine langfristige Verbesserung durch Budesonid, Mycophenolatmofetil und Cyclophosphamid; mit Tofacitinib reduzierte Inflammation und Verbesserung von H, T, D und S. |
| 8 | 79 | W | H, T, D2, S1, Dilation, HP2, FNA | Topisches Budesonid | Reduzierte Entzündungsreaktion, Verbesserung der S, D, T |
| 9 | 83 | W | D1, HP1, FNA | Topisches Budesonid | Keine Reendoskopie in unserem Zentrum |
| 10 | 74 | W | H, T, HP1, F2 | Topisches Budesonid | Keine Reendoskopie in unserem Zentrum |
| 11 | 56 | W | H, T, D1, HP0, F2 | Methotrexat, Mycophenolatmofetil | Reduzierte Entzündungsreaktion, |
| 12 | 63 | W | D1, HP0, F1 | systemisches Glucocorticosteroide, Methotrexat | Reduzierte Entzündungsreaktion, |
| 13 | 87 | M | HP1, F2 | Keine Behandlung | Keine Behandlung |
| 14 | 65 | W | D1, HP2, F2 | Topisches Budesonid, Guselkumab, Mycophenolatmofetil, Baricitinib | Kein Effekt von topischem Budesonid und Mycophenolatmofetil, Guselkumab: sekundärer Wirkverlust, Baricitinib: Verbesserung der Entzündung und D |
| 15 | 87 | W | H, T, S1, HP2. FNA | Topisches Budesonid | Reduzierte Entzündungsreaktion, Verbesserung der S, T |
| 16 | 75 | W | H, T, D1, HP3, FNA | Topisches Budesonid | Keine Reendoskopie in unserem Zentrum |
| 17 | 75 | W | T, D1, S2, HP2, F1 | Topisches Budesonid | Reduzierte Entzündungsreaktion, wiederkehrende Pilz-Ösophagitis, Verbesserung von S, T, D |
| 18 | 62 | W | H, T, D1, S1, HP3, F1 | Topisches Budesonid | Komplette Remission von H, S, T |
| 19 | 79 | M | H, T, HP2, FNA | NA | NA |
| 20 | 45 | W | T, S2, Dilation, HP1, FNA | Mycophenolatmofetil | Keine Reendoskopie in unserem Zentrum |
| 21 | 81 | W | D1, S1, HP1 | Topisches Budesonid | Keine Reendoskopie in unserem Zentrum |

*Abk.:* D, Denudation; DIF, direkte Immunfluoreszenz; F, Fibrinogenablagerung in der DIF; H, Hyperkeratose; HP, Histopathologie; LP, Lichen planus; NA, nicht verfügbar; S, Stenose; T, Trachealisierung
